# Isolation and Genomic Characterization of the G6P[1]-Type Sheep Rotavirus in China

**DOI:** 10.1155/2024/9614599

**Published:** 2024-07-01

**Authors:** Ping Li, DengShuai Zhao, TianYu Wang, DiXi Yu, KeShan Zhang

**Affiliations:** ^1^ School of Life Science and Engineering Foshan University, Foshan 528000, Guangdong, China; ^2^ College of Animal Science and Technology Tarim University, Alar 843300, Xinjiang, China

## Abstract

Rotavirus A (RVA) is a prevalent cause of enteric diarrhea in infants, bovine, pigs, and sheep globally. Currently, the G6P[1]-type rotaviruses are prevalent in sheep or goat in Bangladesh, Turkey, and Uganda. However, this genotype has not been reported in Chinese sheep or goat. Therefore, 12 anal swabs were collected from diarrheal sheep in Gansu Province, China, in 2023 and tested for rotavirus using reverse transcription-polymerase chain reaction (RT-PCR). Pathological sections and immunohistochemistry were used to observe pathological changes and rotavirus antigens in the duodenum, respectively. The sheep rotavirus was isolated in MA-104 cells and characterized through indirect immunofluorescence and transmission electron microscopy. The genes of the strain were obtained using the next-generation sequencing technology and analyzed phylogenetically. One sheep was positive for rotavirus by RT-PCR, and immunohistochemistry revealed numerous rotavirus antigens in the apical portion of the duodenal villi. Transmission electron microscopy revealed that the strain was characterized by virus particles that were “wheel-shaped” and measured 70–80 nm in size. The gene constellations of this strain is G6-P[1]-I2-R2-C2-M2-A11-N2-T6-E2-H3. BLASTn and phylogenetic tree analyses suggest that this strain is likely a recombinant of human rotavirus, goat rotavirus, and bovine rotavirus. The comparison of amino acid similarities revealed three differences in the key antigenic epitopes of the VP7 and VP4 proteins between the GO34 strain and this study strain despite the identical gene constellations of the two strains. To date, this is the first report of this constellation of RVA being found in sheep.

## 1. Introduction

Group A rotavirus (RVA) is known to cause severe gastroenteritis in infants and animals. In developing countries, children under the age of five are seriously threatened by RVA, with over 120,000 children dying each year due to RVA infection [1].

RVA is a member of the rotavirus genus in the Reoviridae family. Its genome is approximately 18,500 bp [2] and consists of 11 segmented dsRNA that encode 6 structural proteins (VP1, VP2, VP3, VP4, VP6, and VP7) and 5/6 nonstructural proteins (NSP1-NSP5/NSP6) [3]. Among these proteins, the protective antigens VP4 (protease-sensitive protein) and VP7 (glycosylated protein), located on the surface of the viral structure, comprise viral capsid proteins and determine the P and G genotypes, respectively [4]. The segmented nature of the RVA genome and the accumulation of mutations, genetic reassortment, interspecies transmission, recombination, and rearrangement result in a rich genetic diversity of this virus [5]. In response to the need to study the genetic diversity of RVs, the classification of RVs has been generalized to the whole genome. In 2008, the Rotavirus Classification Working Group recommended a more comprehensive classification scheme based on the genotypes of all 11 segments: Gx-P[x]-Ix-Rx-Cx-Mx-Ax-Nx-Tx-Ex-Hx [6, 7]. To date, 42 G genotypes, 58 P genotypes, 32 I genotypes, 28 R genotypes, 24 C genotypes, 23 M genotypes, 39 A genotypes, 28 N genotypes, 28 T genotypes, 32 E genotypes, and 28 H genotypes have been reported [3, 8]. Thus, the classification of RV has facilitated our understanding of RV genetic diversity and cross-species transmission.

Numerous deaths of goat and sheep due to diarrhea caused by different genotypes of RVA infection have been reported worldwide. In 2011, an outbreak of ovine diarrheic syndrome was diagnosed in a flock of lambs on a farm in Spain and the G8P[1]-type RVA was found to be the main cause [9]. In 2012, the G6P[11]-RVA was first detected in the diarrheal feces of lamb in India [10]. In our previous study, the G8-type RVA was present in diarrheal sheep [11]. Due to RVA transmission between different hosts, differences in the prevalence of its genotypes exist in different regions. The G6P[1]-type RVA was detected in diarrheal goat in Bangladesh and was closely related to bovine rotavirus, ovine rotavirus, antelope rotavirus, and human rotavirus [12]. The G1P[8]-type and G1P[4]-type RVA, which occur in goat in India, are potentially transmissible to humans [13]. In addition, the human G3P[10]-type RVA, which was detected in India, was also shown to be of probable goat origin [14]. Therefore, goat RVA is a sub-risk factor for cross-species transmission. There are few reports on goat and lamb RVA in China; the RVA isolates LLR [15], Lamb-NT [16], XL (JQ004970-JQ004980), and CC0812-1 [16] all belong to the G10P[15]-type. In this study, the G6P[1]-RVA was detected in anal swabs of diarrheic sheep. The strain was identified, isolated, and genome-wide analyzed. This study was to provide data for the genomic study of RVA in sheep and to provide a basis for the prevention and control of diarrheal diseases in sheep.

## 2. Materials and Methods

### 2.1. Sample Collection

In 2023, a total of 12 anal swabs were collected from lambs with severe diarrhea on farms in Gansu Province, China. Samples were collected in 15 mL centrifuge tubes supplemented 2 mL PBS and stored at low temperature in a sample collection box. Samples were then shipped to the laboratory within 24 hr for storage at −80°C. Reverse transcription-polymerase chain reaction (RT-PCR) was used to screen the samples. The results revealed that one of the samples was infected with RV. The intestinal tubes of this sheep were collected and stored in 4% paraformaldehyde with the farmer's consent to minimize suffering. The intestinal tubes of clinically healthy and RV-negative sheep were collected and stored in the same manner.

### 2.2. Sample RNA Extraction and RT–PCR Amplification

The anal swab samples were frozen and defrosted thrice and centrifuged at 4°C, 12,000 rpm for 10 min. First, 200 *µ*L of total RNA was extracted using TRIzol reagent (Takara, Beijing, China). Second, reverse-transcribe RNA to cDNA using PrimeScript™ RT Master Mix (Perfect, Real Time, Takara Beijing, China). RT−PCR was used for RV detection of cDNA, and primers were synthesized against RV VP6 (Forward primer: 5-GATGTCCTGTACTCCTTGT-3; Reverse primer: 5-GGTAGATTACCAATTCCTCC-3) [17]. The reaction was run in a thermocycler (Bio-Rad) according to the following program: denaturation at 95°C for 3 min, followed by 35 cycles at 95°C for 30 s, 50°C for 30 s, and 72°C for 45 s. Final extension at 72°C for 10 min. The expected product size is 160 bp. Those PCR products were sent to Xi'an Biotechnology for sequence determination.

### 2.3. Histopathology and Immunohistochemistry

The duodenum pathological sections were analyzed by Wuhan Xavier Biotechnology Company using hematoxylin and eosin staining and immunohistochemistry.

### 2.4. Virus Isolation

To enhance the virus's ability to invade cells, we added trypsin (Solarbio, T-1350-100 mL) at a final concentration of 15 *μ*g/mL and 1% penicillin-streptomycin solution (Gibco, 15070063) to the positive samples and allowed to stand for 1 hr at 37°C in a constant temperature water bath.

The well-growing MA-104 cell culture was discarded, and the cell's surface was rewashed three times with PBS. The RV activated by trypsin was added, and the cells were cultivated in the cells culture incubator at 37°C with 5% CO_2_. The cell vial was gently shaken every 30 min to allow the virus to adsorb onto the cells uniformly and sufficiently. After 2 hr, the solution in the cell vial was discarded, and the cell's surface was rewashed three times with PBS. The cell maintenance solution with trypsin at a final concentration of 4 *μ*g/mL was then added, and a negative control group without trypsin was set up. The cells were cultured at 37°C under 5% CO_2_ for 3 days, and cytopathic effect (CPE) was observed. Viral fluids were collected only when CPE was present. The cell culture flasks were then subjected to three rounds of freeze–thawing. Next, the entire liquid was centrifuged at 12,000 rpm/min at 4°C for 10 min, and the supernatant was collected. The virus was then passaged blindly to the 13th generation.

### 2.5. Transmission Electron Microscopy

First, the ultracentrifuge tube was filled by adding 10 mL of the 20% sucrose solution to the bottom. About 150 mL of virus solution was added, and the tube was centrifuged at 170,000× *g* for 4 hr at 4°C. The supernatant was discarded after ultracentrifugation. The virus was resuspended in 1 mL PBS. Second, the l mL virus in sucrose density gradient was centrifuged at 220,000× *g* for 4 hr at 4°C. The desugarization centrifugation at 220,000× *g* for 2 hr at 4°C. Finally, the virus was resuspended in 500 *µ*L PBS and subjected to transmission electron microscopy after treatment with 2% phosphotungstic acid negative stain.

### 2.6. Indirect Immunofluorescence Assay (IFA)

The MA-104 cells were transferred to six-well plates. After the cells have grown and covered approximately 90% of the bottom of the wells, inoculate each well with 100 *µ*L of the virus solution. The negative control well was not added to the virus solution. After cells displayed significant CPE, cells in all wells were fixed with 4% paraformaldehyde solution for 1 hr at room temperature. The cells were permeabilized with 0.1% Triton-X-100 for 30 min at room temperature and then blocked with 5% BSA for 1 hr at room temperature. The anti-RVA-VP6 monoclonal antibody (2B4: Santa Cruz Biotechnology, sc-101363) was used as the primary antibody, and the cells were treated at 37°C for 1 hr. The goat antimouse IgG H&L (Alexa Fluor® 488) (Abcam, ab150113) was used as the secondary antibody, and the cells were treated for 1 hr at 37°C under light protection. The cell nuclei were colored for 7 min with DAPI (King Clone Biotechnology Co. CC1162) at a 1 : 3,000 dilution with PBS. The samples were then observed under an inverted fluorescence microscope.

### 2.7. Next-Generation Sequence of GS2023

The isolate obtained in this study has been named “GS2023.” About 50 *μ*L (220 ng/*μ*L) of RNA extracted from the GS2023 strain was sent to the Shanghai Tanpu Biotechnology Company (Sequencing Platform and Strategy: Illumina Novaseq PE150) in China for the next-generation sequence of the virus. The sequences of the GS2023 strain have been submitted to GenBank under accession numbers PP115426-PP115436.

### 2.8. Genetic and Antigenic Epitopes Analysis

Gene sequences related to this study were downloaded from NCBI and combined with the entire gene sequences obtained by the next-generation sequence in this study for phylogenetic analyzes. MEGA11 (version 11.0.13) software was used to construct phylogenetic trees using the neighbor-joining method.

## 3. Results

### 3.1. Detection of RV in Samples

**Figure 1:**
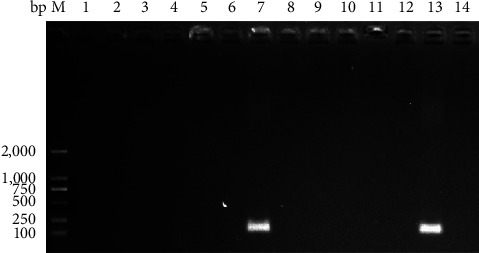
Detection and amplification of the RV VP6 gene. M:DL 2,000 DNA marker. 1–12: anal swab samples from sheep with clinical diarrhea. 13: positive control. 14: negative control.

**Figure 2:**
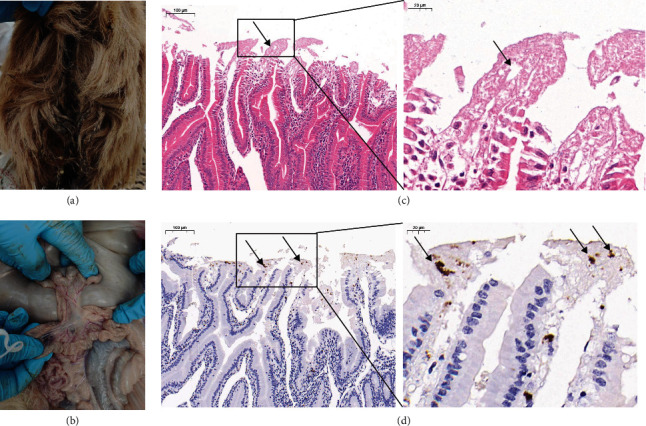
(a) and (b) Display field diagrams of RV-infected sheep. (c) and (d) Display pathological sections and immunohistochemistry of the duodenum of sheep infected with RV.

Out of 12 samples, 1 was positive for RV, resulting in an infection rate of 8.3% (1/12) in sheep with diarrhea (Figure 1). Local abnormalities in the intestine were observed at autopsy, including intestinal wall thinning, intestinal wall hemorrhage, and mesenteric hemorrhage (Figures 2(a) and 2(b)). HE staining of the duodenum revealed autolysis at the tips of the intestinal villi, and immunohistochemical staining revealed localized RV positivity (Figures 2(c) and 2(d)). HE staining (*Supplementary Figure 1(a)*) and immunohistochemistry (*Supplementary Figure 1(b)*) staining of duodenum from RV-uninfected sheep. The available evidence indicates that the sheep were infected with RV.

### 3.2. RV Isolation

**Figure 3:**
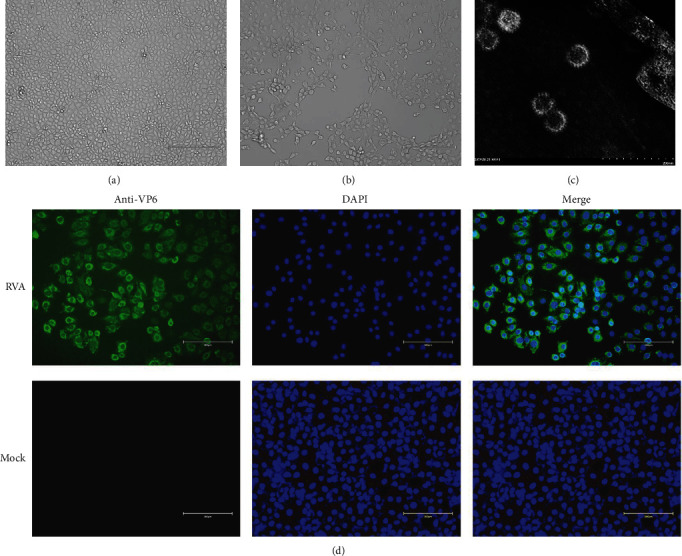
(a) Displays MA-104 cells control. (b) Displays that GS2023 infected MA-104 cells for 12 hr. The morphology of the GS2023 virus particle is shown (c). Detection of the GS2023 infection in MA-104 cells by IFA (d).

RV-positive samples were inoculated into MA-104 cells and passaged. By generation 7, the cells showed a significant CPE. The MA-104 cells that were inoculated with the virus displayed crumpling, pulling, and shedding after 12 hr, while the normal cells exhibited good translucency and clear edges (Figures 3(a) and 3(b)).

Viral particles with a whorled structure were observed in the culture supernatant of RV-inoculated cells using negative staining electron microscopy. The particles were 70–80 nm in size, which is typical of RV virosomes (Figure 3(c)). An IFA using RVA-VP6 monoclonal antibody confirmed the GS2023 strain as RV positive (Figure 3(d)). In summary, the sheep RV (GS2023) was successfully isolated from sheep in China.

### 3.3. Gene Constellations of the GS2023 Strain

The isolated RVA (GS2023) open reading frame sequences were obtained by assembling next-generation sequence reads using de novo methods. The raw data of the next-generation sequencing was uploaded in supplementary materials (*Supplementary 2*). The GS2023 strain has a typical structure and characteristics of the RVA genome. The genome conformational state (GCs) of the GS2023 strain was G6-P[1]-I2-R2-C2-M2-A11-N2-T6-E2-H3 (Table 1).

### 3.4. Genetic Evolutionary Analysis

**Figure 4:**
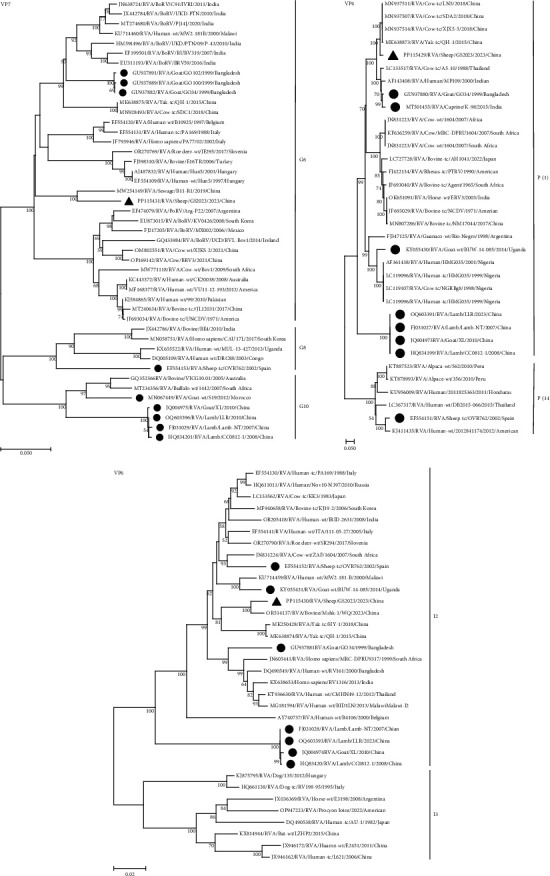
Phylogenetic trees for the VP7 gene, the VP4 gene, and the VP6 gene. The numbers at the nodes indicate the level of bootstrapping based on neighbor-joining analysis of 1,000 replications. Only values above 50% have been shown. Triangles mark strains in this study; circles mark reported goat, lamb, and sheep strains.

**Figure 5:**
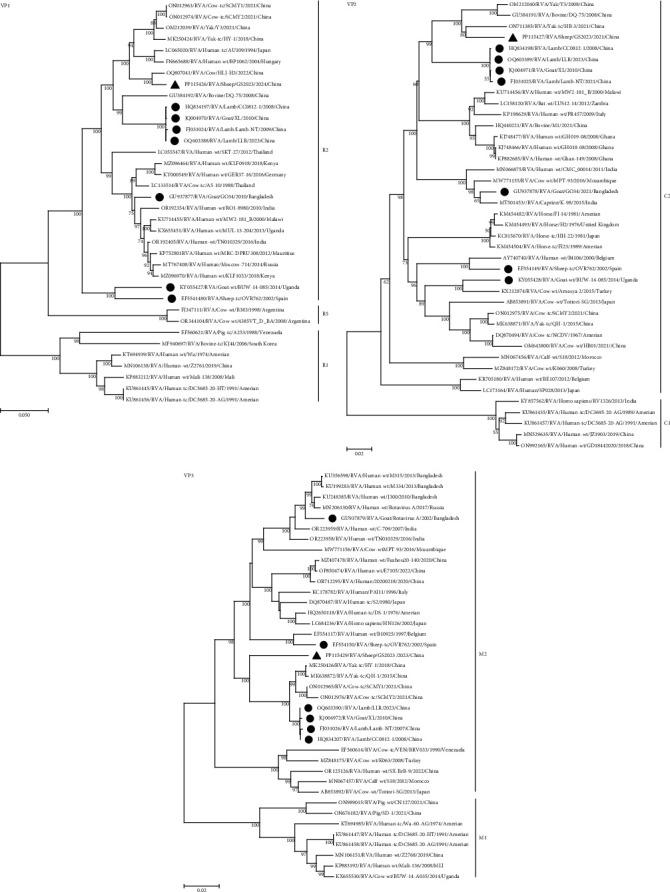
Phylogenetic trees for the VP1 gene, the VP2 gene, and the VP3 gene. The numbers at the nodes indicate the level of bootstrapping based on neighbor-joining analysis of 1,000 replications. Only values above 50% have been shown. Triangles mark strains in this study; circles mark reported goat, lamb, and sheep strains.

**Figure 6:**
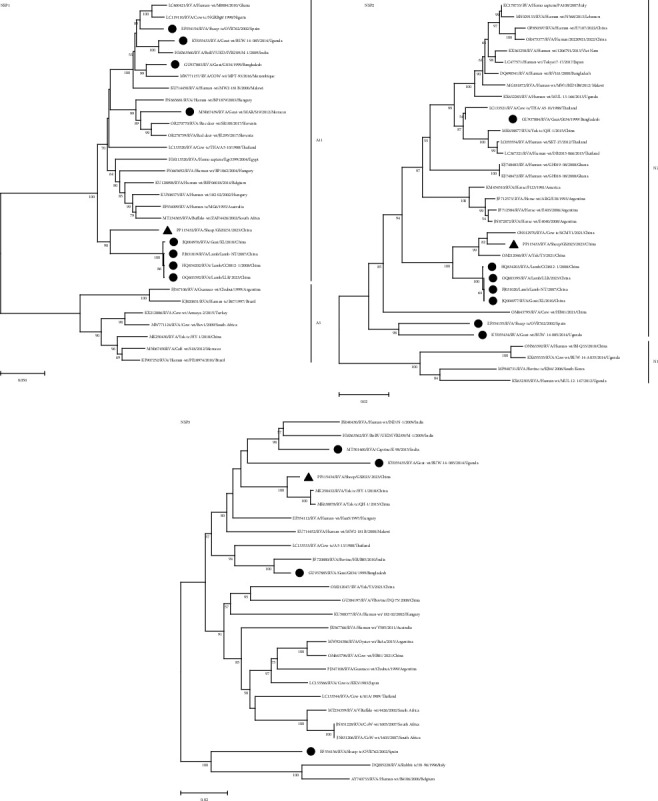
Phylogenetic trees for the NSP1 gene, the NSP2 gene, and the NSP3 (the genotypes of all the sequences in the NSP3 are T6) gene. The numbers at the nodes indicate the level of bootstrapping based on neighbor-joining analysis of 1,000 replications. Only values above 50% have been shown. Triangles mark strains in this study; circles mark reported goat, lamb, and sheep strains.

**Figure 7:**
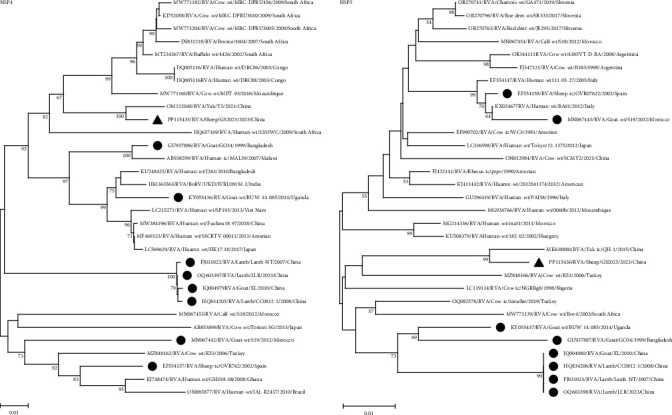
Phylogenetic trees of the NSP4 (the genotypes of all the sequences in the NSP4 are E2) gene and NSP5 (The genotypes of all the sequences in the NSP5 are H3) gene. The numbers at the nodes indicate the level of bootstrapping based on neighbor-joining analysis of 1,000 replications. Only values above 50% have been shown. Triangles mark strains in this stud; circles mark reported goat, lamb, and sheep strains.

Further insight into the origin of the strain is provided by genetic and evolutionary analyses of each of the 11 genes of the GS2023 strain (Figures 4, 5, 6, and 7).

BLASTn showed no sequences of the GS2023 strain were 100% similar to the sequences available in GenBank. Nucleic acid similarity analysis revealed that the VP2 and NSP2 genes had the highest similarity to human RVA, the VP1, VP4, VP6, NSP3, NSP4, and NSP5 genes had the highest similarity to bovine RVA genes, the VP3 and NSP1 genes had the highest similarity to the genes of LLR strains of lamb. The VP7 gene had the highest similarity to found in sewage, indicating a possible relationship between the VP7 gene and the human rotavirus VP7 gene. In Phylogenetic trees, the VP7 gene is closely related to the G6 genotype found in untreated residential sewage from Jinan, China, and is not in the same branch as other G6 genotypes. The VP4 gene is closely related to the P[1] genotype of Chinese cow and yak-origin rotavirus and forms a separate branch. The VP6 gene forms a branch with the I2 genotype of Chinese bovine and yak origin but is most closely related to the I2 genotype of bovine rotavirus. The VP1 gene is most closely related to the R2 genotype of Chinese cow rotavirus. The VP2 gene is closely related to the C2 genotype of Chinese yak rotavirus, bovine rotavirus, lamb rotavirus, and goat rotavirus and forms a separate branch. The VP3 gene forms a branch with the M2 genotype of Chinese yak rotavirus, bovine rotavirus, cow rotavirus, lamb rotavirus, and goat rotavirus. The NSP1 gene forms a separate branch from the A11 genotype of Chinese lamb rotavirus and goat rotavirus. The NSP2 gene is closely related to the N2 gene of cow rotavirus, yak rotavirus, lamb rotavirus, and goat rotavirus in China. The NSP3 gene is most closely related to the T6 genotype of the Chinese yak rotavirus. The NSP4 and NSP5 genes were most closely related to the E2 and H3 genotypes of the Chinese yak rotavirus, respectively, and formed a separate branch in the genetic evolution tree.

The phylogenetic trees showed that the genes of the GS2023 strain were closely related to RVA genes found in bovine rotavirus, lamb rotavirus, goat rotavirus, and residential sewage rotavirus from China. This suggests that the GS2023 strain may share a common origin with RV of bovine, goat, and humans and have the potential for cross-transmission.

### 3.5. Differentiation VP4 and VP7 Antigens Epitopes Analysis between the GO34 Strain and GS2023 Strain

To determine whether there are differences between the GS2023 strain and the GO34 strain, we analyzed the major antigenic epitope differences on the surface of VP7 and VP4 proteins in these two strains. The GS2023 strain showed three amino acid differences compared with the GO34 strain, including N238D (7-1b region), A242T (7-1b region), and T440M (5-1 region) (Tables 2 and 3).

## 4. Discussion

Humans and various animals can be infected with RVA, causing clinical disease [18]. Currently, there is a gap in the reports describing the morphology of the intestine and the pathological changes in the intestine after RV infection in sheep. Infection of suckling mice with bovine RVA resulted in mild hemorrhage in the small intestine. HE staining of the jejunum and ileum showed vacuolation and pyknosis of the nuclei of mature enterocytes, as well as their lysis and detachment. Villi constriction and detachment were also observed, along with mild mononuclear cell infiltration in the lamina propria and mild cell depletion of Peyer's patches and mesenteric lymph nodes (MLN) [19]. HE microscopy of the G9P[23] type RVA and the G9P[7] type RVA infected piglets revealed small intestinal villous atrophy and crypt hyperplasia [18]. The strain CN127 was found to cause edema of the intestinal canal and epithelial detachment of the jejunal villi after infection in piglets [3]. The SA11, 16-06, and MpR12 strain-infected mice showed vacuolization of enterocytes on most of the villous surface and increased inflammatory cell infiltration in the lamina propria [20]. According to these previous findings, it can be concluded that there are variations in the pathological changes that occur following different RV infections. In this study, we found thinning of the intestinal wall, intestinal wall hemorrhage, mesenteric hemorrhage, and autolysis of the duodenal enterochromaffin epithelium in RVA-infected sheep, but we did not find pathological changes, such as vacuolization of enterocytes, which are characteristic of RVA infection. However, immunohistochemistry revealed RV-positive signals in the duodenum, thus demonstrating that the sheep were infected with RV. Further animal experiments are necessary to determine if the autolysis of the duodenal villous epithelium observed in the RVA-infected sheep in this study was caused by RVA.

In the process of RV isolation, trypsin is required to cleave the VP4 protein into VP5 ^*∗*^ and VP8 ^*∗*^ to facilitate the entry of RV into susceptible cells [2, 21]. Therefore, we used EDTA-free trypsin to activate RV during RV isolation and serum-free medium with a certain trypsin concentration. After the 10th, the cells began to show stable CPE, including crumple, pull, and shed. When observing the morphology of the RV viral particles via transmission electron microscopy, the particles were wheel-shaped and had a diameter of approximately 70–80 nm, which is consistent with the size reported by previous studies [22]. We also successfully verified the expression of the RVA antigen in MA-104 cells using an RV-VP6 monoclonal antibody, which confirmed that the GS2023 strain was successfully isolated.

Because the large and rich diversity of the RV genome results in the absence of shared primers for RT–PCR [20]. To understand the characterization of the GS2023 strain, we have chosen next-generation sequencing. The GCs of GS2023 strain was G6-P[1]-I2-R2-C2-M2-A11-N2-T6-E2-H3. This is the first time that this constellation of RVA has been detected in sheep in China. Comparison of the sequencing results with the uploaded sequences in GenBank revealed that the VP2 and NSP2 genes showed the highest similarity to human RV genes, the VP1, VP4, VP6, NSP3, NSP4, and NSP5 genes showed the highest similarity to bovine rotavirus genes, the VP3 and NSP1 genes showed the highest similarity to the genes of LLR strains from sheep, and the VP7 genes showed the highest similarity to the RV VP7 gene found in sewage. In the VP7 phylogenetic tree, the GS2023 strain was found to be closely related to the RVA/sewage/CHN/B11-R1/2019/G6 from residential sewage in Jinan, China [23]. However, our sampling site was approximately 1,390 km away from Jinan City. The sheep with diarrhea in the homes of the farmers surveyed in this study often have access to sewage generated by local residents. Therefore, we suspected that the sewage produced by the local population contained rotavirus type G6. Of course, more research is needed to prove this assumption. In other phylogenetic trees, the GS2023 strain was found to be related to Chinese bovine rotavirus, lamb rotavirus, and goat rotavirus. In conclusion, the GS2023 strain was closely related to rotaviruses of bovine, goat, and humans in China.

The lamb RVA isolates LLR, Lamb-NT, XL, and CC0812-1 found in China, were all identified as G10P[15]. To date, only diarrheic goat from Bangladesh [12] and asymptomatic goat from Uganda [24] have been reported to have G6-P[1]-I2-R2-C2-M2-A11-N2-T6-E2-H3 genotype RVA (Table 4). The Bangladeshi goat GO34 strain and the Uganda RVA/Goat-wt/UGA/BUW-14-085/2014/G6P[1] strain were genetically identical to the GS2023 strain. This study identified the presence of this genotype in sheep. Therefore, the G6-P[1]-I2-R2-C2-M2-A11-N2-T6-E2-H3 genotype can infect both sheep and goat. The Uganda RVA/Goat-wt/UGA/BUW-14-085/2014/G6P[1] strain was found in asymptomatic goat. However, the GO34 and GS2023 strains were derived from goat and sheep exhibiting diarrheal symptoms, respectively. Thus, the GO34 strain and the GS2023 strain may be the pathogens causing diarrhea. Altering the structure of key antigens may affect the infectivity and antigenic properties of the viruses [3]. The VP7 and VP4 genes encode the outer proteins of RVA and play an important role in inducing neutralizing antibodies [25]. The major antigenic epitopes of VP7 are exposed in 7-1 (7-1a, 7-1b) and 7-2 regions [25], and the major antigenic epitopes of VP4 are exposed in 5-1, 5-2, 5-3, 5-4, 5-5, 8-1, 8-2, 8-3, and 8-4 regions [26]. To anticipate if the antigenic properties and infectivity of the GO34 strains and the GS2023 strains are equivalent. Therefore, we analyzed the amino acid changes in the major antigenic epitopes of VP7 and VP4 to identify the differences between the GO34 strain and the GS2023 strain. Only three amino acids were found to be changed in the 7-1b region and in the 5–1 region. These changes may affect the infectivity and antigenic properties of the viruses.

Diarrhea is a significant contributor to the mortality rate of lambs [27]. Rotavirus in sheep/goat should be of high concern. It is a common cause of diarrhea worldwide. In addition, pathogenetic studies help to understand its origin and spread [28]. Although there is no commercial sheep RV vaccine available in China, this study is beneficial in providing a basis for future vaccine research and development.

## 5. Conclusions

The GS2023 strain was successfully isolated, and its constellation was G6-P[1]-I2-R2-C2-M2-A11-N2-T6-E2-H3. The GS2023 strain was identified as a recombinant of bovine rotavirus, goat rotavirus, and human rotavirus. To date, this is the first report of this constellation of RVA being found in sheep. This result will contribute to the diagnosis and prevention of diarrhea in sheep, as well as improving our understanding of rotaviruses in sheep.

## Figures and Tables

**Table 1:** Genotype constellation of the GS2023.

Gene	Genotype	Strain exhibiting the highest identity^a^	Accession no.	Nucleotide identity (%)	Cutoff (%)^b^
		Strain name			
VP1	R2	RNA/Bovine/HLJ-H3/2022/CHN/G6P[5]-R2	OQ807041	97.34	83
VP2	C2	RVA/Human-wt/HUN/BP1062/2004/G8P[14]-C2	FN665689	94.13	84
VP3	M2	Lamb rotavirus strain LLR/ G10P[15]-M2	OQ603390	90.63	81
VP4	P[1]	RVA/Cow-tc/CHN/XJX5-5/2018/G6P[1]-P[1]	MN937514	96.85	80
VP6	I2	Mshk-1/WQ/Xinjiang/China/2023/G8P[X]-I2	OR514137	98.14	85
VP7	G6	RVA/sewage/CHN/B11-R1/2019/G6-G6	MW254149	92.46	80
NSP1	A11	Lamb rotavirus strain LLR/ G10P[15]-A11	OQ603392	89.88	79
NSP2	N2	RVA/Human-wt/VNM/NT0578/2008/G2P[4]-N2	LC060821	97.09	85
NSP3	T6	RVA/Yak-tc/CHN/HY-1/2018/G6P[11]-T6	MK250432	98.58	85
NSP4	E2	Y3/G6P[5]-E2	OM212048	97.94	85
NSP5	H3	RVA/Yak-tc/CHN/QH-1/2015/G6P[1]-H3	MK638880	97.94	91

^a^Closest strains were identified using BLASTn on megablast setting. ^b^Matthijnssens et al. [6, 7].

**Table 2:** Alignment of the amino acid residues in VP4 antigenic epitopes.

	8-1											8-2		8-3									8–4		
VP8	100	146	148	150	188	190	192	193	194	195	196	180	183	113	114	115	116	125	131	132	133	135	87	88	89
GO34	N	Q	Q	G	Y	S	T	N	Y	D	S	E	N	Q	S	V	E	Q	S	N	D	Q	T	N	A
GS2023	N	Q	Q	G	Y	S	T	N	Y	D	S	E	N	Q	S	V	E	Q	S	N	D	Q	T	N	A
	5-1								5-2	5-3	5-4	5-5													
VP5	384	386	388	393	394	398	440	441	434	459	429	306													
GO34	D	S	A	N	Y	T	T	T	E	Q	R	T													
GS2023	D	S	A	N	Y	T	M	T	E	Q	R	T													

**Table 3:** Alignment of the amino acid residues in VP7 antigenic epitopes.

	7-1a														7-1b					
VP7	87	91	94	96	97	98	99	100	104	123	125	129	130	291	201	211	212	213	238	242
GO34	V	N	A	T	E	W	K	N	C	D	A	V	D	K	C	D	P	N	N	A
GS2023	V	N	A	T	E	W	K	N	C	D	A	V	D	K	C	D	P	N	D	T
	7-2																			
VP7	143	145	146	147	148	190	217	221	264											
GO34	K	D	S	T	L	S	T	T	G											
GS2023	K	D	S	T	L	S	T	T	G											

**Table 4:** Comparison of genotypes of different strains.

Strain	Host	Country	VP1	VP2	VP3	VP4	VP6	VP7	NSP1	NSP2	NSP3	NSP4	NSP5
GS2023	Sheep	China	R2	C2	M2	P[1]	I2	G6	A11	N2	T6	E2	H3
GO34	Goat	Bangladesh	R2	C2	M2	P[1]	I2	G6	A11	N2	T6	E2	H3
RVA/Goat-wt/UGA/BUW-14-085/2014/G6P[1]	Goat	Uganda	R2	C2	M2	P[1]	I2	G6	A11	N2	T6	E2	H3
LLR	Lamb	China	R2	C2	M2	P[15]	I2	G10	A11	N2	T3	E2	H3
Lamb-NT	Lamb	China	R2	C2	M2	P[15]	I2	G10	A11	N2	T3	E2	H3
CC0812-1/2008	Lamb	China	R2	C2	M2	P[15]	I2	G10	A11	N2	T3	E2	H3
XL	Goat	China	R2	C2	M2	P[15]	I2	G10	A11	N2	T3	E2	H3
RVA/Goat-wt/TUR/Eskisehir-1/2009/G6P[1]	Goat	Turkey	X	X	X	P[1]	I2	G6	X	X	X	E2	X

GenBank numbers: GS2023: PP115426-PP115436, LLR: OQ603388-OQ603398, Lamb-NT: FJ031019-FJ031029, XL: JQ004970-JQ004980, CC0812-1/2008: HQ834197-HQ834207, RVA/Goat-wt/UGA/BUW-14-085/2014/G6P[1]: KY055427-KY055437, RVA/Goat-wt/TUR/Eskisehir-1/2009/G6P[1]: JX131346, JQ993409, JX076841, JQ956396.

## Data Availability

We are committed to the truthfulness and reliability of the data in this study.

## Supplementary Materials

Supplementary 1 Figure 1: pathological sections (a) and immunohistochemistry (b) of duodenum from RV-uninfected sheep.

Supplementary 2 Raw data of the next-generation sequencing.

## References

[1] Clark A., Black R., Tate J. (2017). Estimating global, regional and national rotavirus deaths in children aged <5 years: current approaches, new analyses and proposed improvements. *PlOS ONE*.

[2] Desselberger U. (2014). Rotaviruses. *Virus Research*.

[3] Miao Q., Pan Y., Gong L. (2022). Full genome characterization of a human-porcine reassortment G12P[7] rotavirus and its pathogenicity in piglets. *Transboundary and Emerging Diseases*.

[4] Matthijnssens J., Ciarlet M., Rahman M. (2008). Recommendations for the classification of group A rotaviruses using all 11 genomic RNA segments. *Archives of Virology*.

[5] Matthijnssens J., Heylen E., Zeller M., Rahman M. A., Lemey P., Van Ranst M. (2010). Phylodynamic analyses of rotavirus genotypes G9 and G12 underscore their potential for swift global spread. *Molecylar Biology and Evolution*.

[6] Matthijnssens J., Ciarlet M., Heiman E. (2008). Full genome-based classification of rotaviruses reveals a common origin between human Wa-Like and porcine rotavirus strains and human DS-1-like and bovine rotavirus strains. *Journal of Virology*.

[7] Matthijnssens J., Ciarlet M., McDonald S. (2011). Uniformity of rotavirus strain nomenclature proposed by the Rotavirus Classification Working Group (RCWG). *Archives of Virology*.

[8] Park G.-N., Kim D. I., Choe S. E. (2022). Genetic diversity of porcine group A rotavirus strains from pigs in South Korea. *Viruses*.

[9] Galindo-Cardiel I., Fernández-Jiménez M., Luján L. (2011). Novel group A rotavirus G8P[1] as primary cause of an ovine diarrheic syndrome outbreak in weaned lambs. *Veterinary Microbiology*.

[10] Gazal S., Taku A. K., Kumar B. (2012). Predominance of rotavirus genotype G6P[11] in diarrhoeic lambs. *The Veterinary Journal*.

[11] Li P., Gai W., Zhao D. (2024). Isolation and molecular characterization of the firstG8-type sheep rotavirus identified in China. https://assets.researchsquare.com/files/rs-3788278/v1/24330547-73-4b83-8092-ec20c3fc261f.pdf?c=1704192131.

[12] Ghosh S., Alam M. M., Ahmed M. U., Talukdar R. I., Paul S. K., Kobayashi N. (2010). Complete genome constellation of a caprine group A rotavirus strain reveals common evolution with ruminant and human rotavirus strains. *Journal of General Virology*.

[13] Choudhary P., Prasad M., Ranjan K., Basanti B. (2017). Zooanthroponotic transmission of rotavirus in Haryana state of northern India. *Acta Virologica*.

[14] Mukherjee A., Mullick S., Kobayashi N., Chawla-Sarkar M. (2012). The first identification of rare human group A rotavirus strain G3P[10] with severe infantile diarrhea in eastern India. *Infection, Genetics and Evolution*.

[15] Li D., Xu Z., Xie G. (2015). Genotype of rotavirus vaccine strain LLR in China is G10P[15]. *Chinese Journal of Virology*.

[16] Yang J., Wang S., Tian L. (2012). Development of neutralizing monoclonal antibodies against VP4 of rotavirus CC0812-1. *Hybridoma*.

[17] Soares V. M., dos Santos E. A. R., Tadielo L. E. (2022). Detection of adenovirus, rotavirus, and hepatitis E virus in meat cuts marketed in Uruguaiana, Rio Grande do Sul, Brazil. *One Health*.

[18] Kim H.-H., Park J.-G., Matthijnssens J. (2013). Pathogenicity of porcine G9P[23] and G9P[7] rotaviruses in piglets. *Veterinary Microbiology*.

[19] Kashyap G., Singh R., Malik Y. S. (2018). Experimental bovine rotavirus-A (RV-A) infection causes intestinal and extra-intestinal pathology in suckling mice. *Microbial Pathogenesis*.

[20] Kishimoto M., Kajihara M., Tabata K. (2023). Isolation and characterization of distinct rotavirus A in bat and rodent hosts. *Journal of Virology*.

[21] Reslan L., Mishra N., Finianos M. (2020). The origins of G12P[6] rotavirus strains detected in Lebanon. *Journal of General Virology*.

[22] Tacharoenmuang R., Guntapong R., Upachai S. (2021). Full genome-based characterization of G4P[6] rotavirus strains from diarrheic patients in Thailand: evidence for independent porcine-to-human interspecies transmission events. *Virus Genes*.

[23] Du H., Xiong P., Ji F. (2021). Genetic diversity and molecular epidemiological characterization of group A rotaviruses in raw sewage in Jinan by next generation sequencing. *Infection, Genetics and Evolution*.

[24] Bwogi J., Jere K. C., Karamagi C. (2017). Whole genome analysis of selected human and animal rotaviruses identified in Uganda from 2012 to 2014 reveals complex genome reassortment events between human, bovine, caprine and porcine strains. *PLOS ONE*.

[25] Aoki S. T., Settembre E. C., Trask S. D., Greenberg H. B., Harrison S. C., Dormitzer P. R. (2009). Structure of rotavirus outer-layer protein VP7 bound with a neutralizing fab. *Science*.

[26] Dormitzer P. R., Nason E. B., Prasad B. V. V., Harrison S. C. (2004). Structural rearrangements in the membrane penetration protein of a non-enveloped virus. *Nature*.

[27] Fesseha H., Gebremichael G., Asefa I., Edaso T. (2023). Study on incidence of lamb morbidity and mortality and associated risk factors in the mixed crop-livestock production system of Gewata DistrictKaffa zone, southwestern Ethiopia. *Animal Diseases*.

[28] Cui M., Shen B., Fu Z. F., Chen H. (2022). Animal diseases and human future. *Animal Diseases*.

