# Supplementary material for: Isolation and Genomic Characterization of the G6P[1]-Type Sheep Rotavirus in China
Source: Transbound Emerg Dis. 2024 Jul 1;2024:9614599. doi: 10.1155/2024/9614599 (PMC12016906; doi:10.1155/2024/9614599)

Figure S1. Pathological sections (A) and immunohistochemistry (B) of duodenum from RV-uninfected sheep.

A


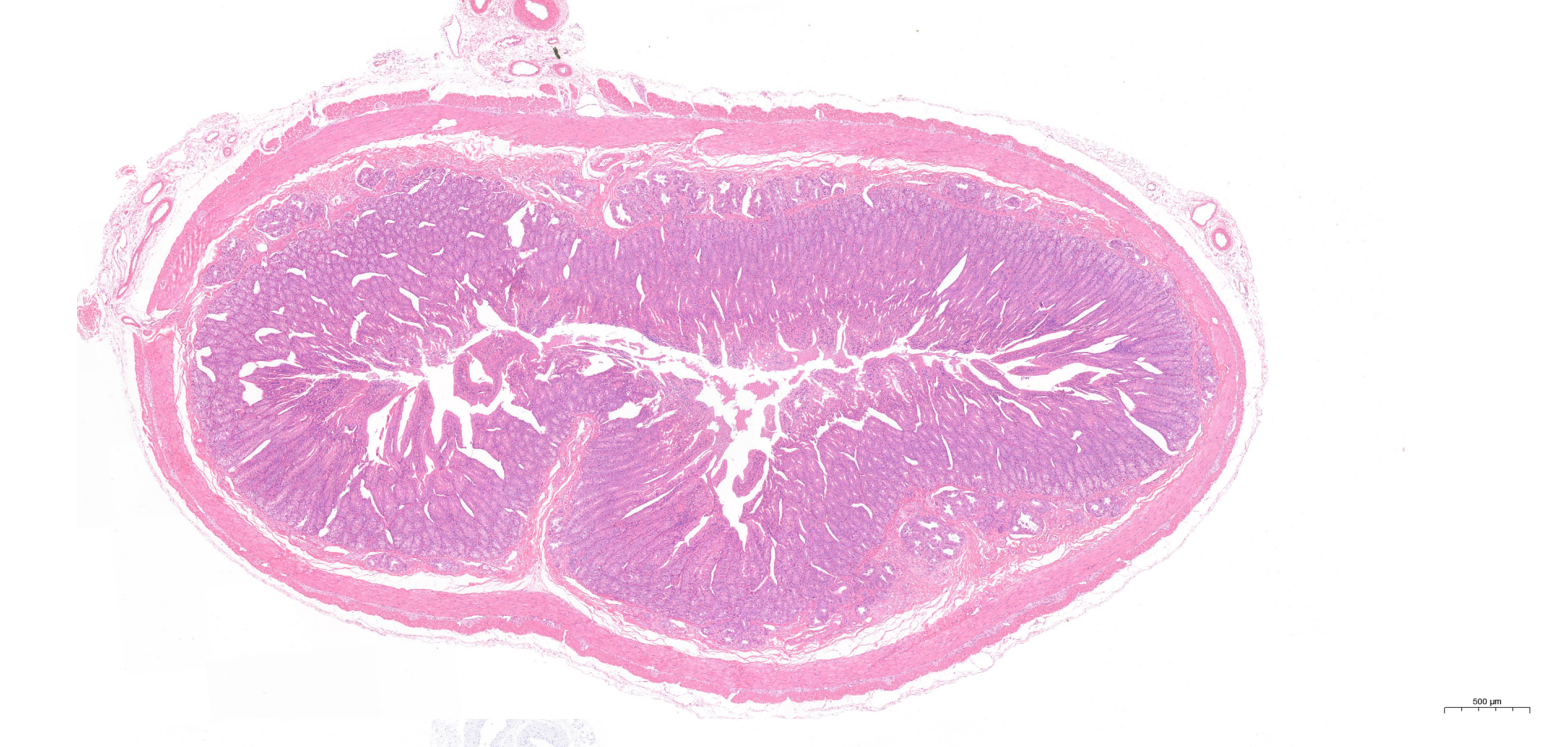


B
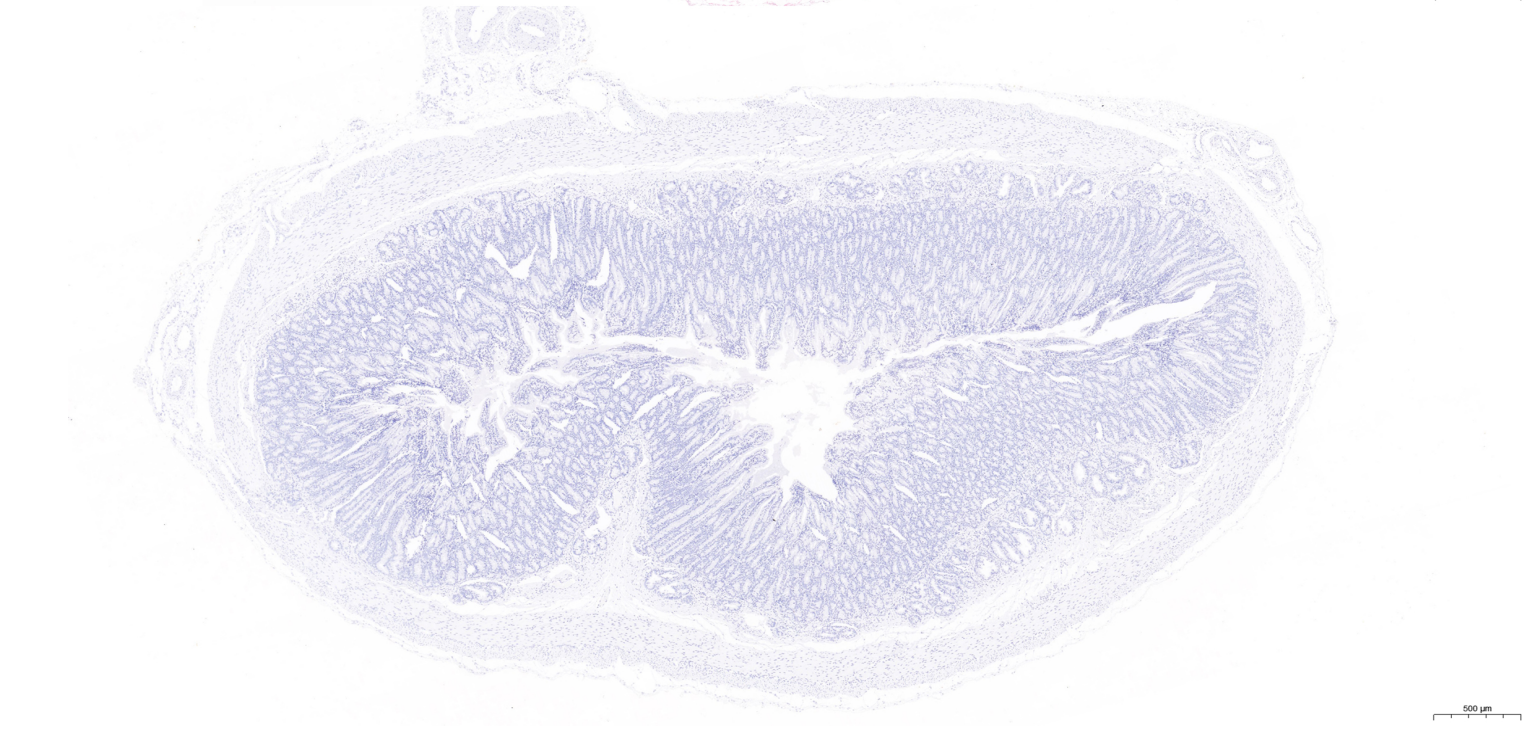

Supplement: Supplementary 1 — Figure 1: pathological sections (a) and immunohistochemistry (b) of duodenum from RV-uninfected sheep. [file 9614599.f1.doc]
